# *Gynura procumbens* Standardised Extract Reduces Cholesterol Levels and Modulates Oxidative Status in Postmenopausal Rats Fed with Cholesterol Diet Enriched with Repeatedly Heated Palm Oil

**DOI:** 10.1155/2019/7246756

**Published:** 2019-09-23

**Authors:** Khuzaidatul Azidah Ahmad Nazri, Norsyahida Mohd Fauzi, Fhataheya Buang, Qodriyah Haji Mohd Saad, Khairana Husain, Ibrahim Jantan, Zakiah Jubri

**Affiliations:** ^1^Department of Biochemistry, Faculty of Medicine, The National University of Malaysia Medical Centre (UKMMC), Kuala Lumpur, Malaysia; ^2^Drug and Herbal Research Centre, Faculty of Pharmacy, The National University of Malaysia Campus Kuala Lumpur, Kuala Lumpur, Malaysia; ^3^Department of Pharmacology, Faculty of Medicine, The National University of Malaysia Medical Centre (UKMMC), Kuala Lumpur, Malaysia; ^4^School of Pharmacy, Taylor's University, Lakeside Campus, 47500 Subang Jaya, Selangor, Malaysia

## Abstract

*Gynura procumbens* (Lour.) Merr. (GP) has been reported in previous studies to possess antihyperlipidaemic, antioxidative, and cardioprotective properties. This study was aimed to determine the effect of standardised 80% ethanol extract of GP on lipid profiles and oxidative status of hypercholesterolemic rats. Postmenopausal (PM) Sprague-Dawley rats were ovariectomised and fed with 2% cholesterol diet fortified with five times heated palm oil to develop hyperlipidaemia status. Two doses of the extract (250 and 500 mg/kg) and atorvastatin (10 mg/kg) were administered once daily via oral gavage for 24 weeks. Systolic blood pressure (SBP) was increased during the first month in the postmenopausal group and decreased with GP supplementation. Lipid droplets accumulation was shown at the tunica media (TM) area of the aorta in the postmenopausal group and reduced with GP supplementation. Total cholesterol (TC), total triglycerides (TG), low-density lipoprotein (LDL), and malondialdehyde (MDA) levels increased (*p* < 0.05) at 3 and 6 months in the postmenopausal group and were reduced with GP supplementation. GP also increased high-density lipoprotein (HDL) level in the postmenopausal group. Superoxide dismutase (SOD), catalase (CAT), and glutathione peroxidase (GPx) activities were reduced in the postmenopausal group compared to control in the sham group but increased (*p* < 0.05) with GP supplementation. The results showed that the higher dose of GP (500 mg/kg) gave better effect. GP has the ability to reduce oxidative stress and prevent membrane cell damage through antioxidant enzyme activity modification and lipid profile changes in postmenopausal rats related to atherosclerosis.

## 1. Introduction

The World Health Organization (WHO) reported that cardiovascular diseases (CVD) are the leading cause of death globally. The National Health and Morbidity Survey of Malaysia reported in 2015 that at least 63% of adults aged 18 years and above had at least one CVD risk factor [1]. The Malaysian Ministry of Health has speculated that CVD incidents will increase to 23.3 million cases in 2030 [2]. In comparison between women and men at matched-age, women have lower risk for cardiovascular diseases especially atherosclerosis. However, when women reached menopause, the incidence of atherosclerosis increases drastically [3]. Studies reported that menopausal women at the age of 55 and above are more prone to develop atherosclerosis [4, 5]. Oestrogen deficiency in women or postmenopausal women has been linked to the rapid increase in CVD [6]. Postmenopause is a condition when the menstrual cycle permanently ceases due to natural depletion of ovarian oocytes due to aging. Thus, oestrogen is no longer produced by the ovary. Oestrogen has a cardioprotective effect towards CVD and atherosclerosis condition [7]. In addition, it increases abdominal fat, triglycerides (TG), total cholesterol (TC), and low-density lipoproteins (LDL) [8, 9]. Hence, it leads to an early atherosclerosis event.

Atherosclerosis is a cardiovascular disease (CVD) that commonly affects the arteries, resulting in plaque formation within the walls of blood vessels. As a result, blood vessel walls become narrowed, impeding blood flow and oxygen delivery, thus causing heart disease [10]. For several years, morbidity, mortality, and hospitalisation rates of atherosclerosis have been persistently in the top five ranks [11]. Many different theories have been used to explain the progression of atherosclerosis, and one of them is oxidative stress. Previous studies have shown the relationship between oxidative stress and atherosclerosis [12]. Oxidative stress is a condition where an imbalance exists between the antioxidant dependence system and the production of reactive oxygen species (ROS), causing lipid peroxidation and free radical formation [13]. Risk factors that attribute to atherosclerosis include hypercholesterolemia, hypertension, cigarette smoking, obesity, sedentary activity, age, family history, diabetes, and gender [14]. In addition, dietary factors such as excessive intake of saturated fatty acids also contribute to the occurrence of atherosclerosis [15]. Currently, lifestyle modification is the first method of treating atherosclerosis while medicine is the next step, where statins are the most popular and widely prescribed drug. However, there are issues pertaining to the usage of statins such as escalating cost and side effects, especially elevated liver enzymes and skeletal muscle damage or myopathy [16]. Thus, new strategies are needed for the prevention of atherosclerosis. Among the best existing alternative therapies are herbal remedies, which have been used since ancient times to treat CVDs.

*Gynura procumbens* (Lour.) Merr locally known as “pokok sambung nyawa” is commonly found in Malaysia, Indonesia, Thailand, Vietnam, and China. It is traditionally used to treat various diseases including fever, rashes, constipation, kidney disease, and cancer [17, 18]. Previous pharmacological studies have shown that *G. procumbens* possesses anti-inflammatory, antihypertensive, antihyperlipidaemic, antioxidative, and cardioprotective properties [19]. To the best of our knowledge, there has been no study yet on the effect of *G. procumbens* on atherosclerosis. The present study was conducted to determine the effects of 80% ethanol extract of *G. procumbens* on lipid profiles and oxidative status in the postmenopausal rat group.

## 2. Materials and Methods

### 2.1. Plant Material

The whole plants of *G. procumbens* were collected from three different locations in Peninsular Malaysia, i.e., Sungai Buloh, Selangor (GP1), Seberang Perai, Pulau Pinang (GP2), and Semenyih, Selangor (GP3). The plants were identified and deposited at the Herbarium of Universiti Kebangsaan Malaysia (UKM), Bangi, Malaysia, with voucher numbers UKMB40375 (GP1), UKMB40378 (GP2), and UKMB40411 (GP3). From 50 kg of fresh whole plants of GP3, 1900 g of powdered dried plant material was obtained and it was macerated with 80% ethanol at a ratio of 1 :  20 (w/v) for 24 h, and the process was repeated thrice per day with occasional stirring. The extract was then filtered through Whatman No. 1 filter paper (Whatman, England). The filtrates were pooled, and excess solvent was removed using a rotary evaporator at 55°C to obtain a dark green extract (92 g). The extract was stored at −4°C for further use. No assessment on heavy metals, pesticides, and other toxic substances was carried out.

### 2.2. Liquid Chromatography-Tandem Mass Spectrometry (LC-MS/MS) Analysis

Liquid chromatography-tandem mass spectrometry (LC-MS/MS) analysis was performed to profile the compounds in the extract. Chromatographic separation was carried out to profile the secondary metabolites of the extract by using the UHPLC system Perkin Elmer Flexar FX15 UHPLC system coupled to Sciex 3200 hybrid trap triple quad tandem mass spectrometer (UHPLC-MSMS), equipped with an autosampler and a Phenomenex Synergy RP C18 column (100 A, 100 × 2.0 mm, 3 *μ*M). The mobile phases were water with 0.1% formic acid (A) and acetonitrile with 0.1% formic acid (B). The detection mode used was targeted multiple reaction monitoring (MRM) for qualification (negative electrospray ionization mode), and the detected compound peaks were compared with mass spectral library (ACD Labs, Toronto, Canada) advanced chemometric mass fragmentations predictive software. LC-MS/MS full chromatograms of *G. procumbens* and chlorogenic acid are shown in [Supplementary-material supplementary-material-1].

### 2.3. Standardisation of 80% Ethanol Extract of *Gynura procumbens* by HPLC

The stock solutions of 80% ethanol extracts of *G. procumbens* (GP1, GP2, and GP3) were each prepared in methanol at 20 mg/ml, and the reference standard, chlorogenic acid solution, was prepared at 1 mg/ml. Each stock solution was then diluted (two-fold dilution) into a series of concentrations. The diluted solutions of the extracts and the reference standards was analyzed separately by HPLC using the following conditions: analytical column XBridge™ C18 (4.6 × 250 mm, 5 mm) was used on Waters 2535 Quartenary Gradient Module with a photodiode array detector (Waters 2998) of wavelength ranging from 210 to 350 nm and Waters Prep Degasser. The mobile phase consisted of acetonitrile (A) and 0.1% orthophosphoric acid (B), with a stepwise gradient system (10% A–90% B at 0 min, 30% A–70% B at 30 min, 100% A at 40 min, and 100% A at 70 min) with a flow rate of 1 ml/min, and injection volume was 10 *μ*L. The identification and quantification of chlorogenic acid in the extracts was performed by comparing the retention time and UV-Vis spectra of the sample peaks with the standard peak (chlorogenic acid) and by plotting a calibration curve of chlorogenic acid, respectively.

#### 2.3.1. Validation Study

The HPLC method was validated by using chlorogenic acid as the external standard. The validation of the HPLC analysis was carried out according to the ICH guidelines [20]. The validation study included precision, linearity, detection limit (LOD), and quantitation limit (LOQ). The precision was investigated at two levels: repeatability (the precision under the same method protocol over a short interval of time or intra-assay precision) and intermediate precision (evaluation of method performance on different days or inter-day precision). Three concentrations of the standard solution, chlorogenic acid (3.91, 31.25, and 250 *μ*g/ml), were injected three times for each concentration in one day (intraday) and on three different days (interday). The calibration curves were established by a series of concentrations (250, 125, 62.5, 31.25, 15.625, 7.8125, and 3.90625 *μ*g/ml) of the standard in triplicate. The calibration curve was constructed by plotting the corresponding peak areas (also known as responses) against the concentrations of the injected standards. The linearity was evaluated by using regression parameters from the calibration curve and correlation coefficient (*R*^2^), calculated from the calibration curves. LOD and LOQ were calculated from the RSD (residual standard deviation) and slope (*S*) of the calibration curves using

(1) $$\begin{array}{c} \text{LOD}=3.3\times \left(\frac{\text{RSD}}{S}\right)\text{,}\\ \text{LOQ}=10\times \left(\frac{\text{RSD}}{S}\right). \end{array}$$

### 2.4. Animals

Forty-eight female Sprague-Dawley rats (2-3 months) weighing 250–300 g were used in this study. The animals were obtained from the Animal House Facility, UKM. All procedures in handling animals were in accordance with the UKM Animal Ethics Guidelines (ethical approval number: BIOK/PP/2016/ZAKIAH/28-SEPT./779-SEPT.-2016-JAN.-2019). The animal management and procedures were performed as recommended in the guidelines. The rats were kept in plastic cages and maintained at room temperature with a 12-h light-dark cycle. All rats were given free access to food and water *ad libitum* during the study period. The rats were allowed to acclimatise for one week prior to the treatment.

### 2.5. Source and Preparation of Diet

Diet used in this experiment contained ground 2% cholesterol pellets and 15% (w/w) of five-time heated palm oil (5HPO). Briefly, 1 kg of sliced sweet potatoes was fried in 2.5 L palm oil (Cap Buruh, Malaysia) in a stainless-steel wok at 180°C for 15 min. The fried sweet potatoes were discarded, and the hot oil was then left to cool at room temperature for 5 h before frying a new fresh batch of sliced sweet potatoes without adding a fresh palm oil. This procedure was repeated four times to obtain 5HPO, following a previous method by Owu et al. [21]. Later, 2% cholesterol diet was ground to powder form and fortified with 15% (w/w) of the prepared 5HPO and then reformed into pellets and dried at 70°C overnight in an oven.

### 2.6. Experimental Protocol

The rats were randomly divided into four sham groups and four postmenopausal (PM) groups, consisting of six rats per group. The postmenopausal group rats were given general anaesthesia and subjected to ovariectomy with some modifications [22]. Briefly, the rats were anaesthetized with a combination of ketamine (80 mg/kg) and xylazine (10 mg/kg) in 1 : 1 ratio based on 0.1 mL per 100 g of body weight. Ovariectomy was preceded by a transverse peritoneal incision of 0.4 to 0.6 cm with a surgical blade on the middle part of the abdomen before the ovary was identified and removed. A braided suture was performed at the sectioned area. The rats in sham groups underwent similar anaesthesia and surgical procedures without removing the ovaries. After surgery, all rats were kept individually. At the same time, iodine was sprayed on the stitched area, and 0.1 mL of antibiotic (Baytril® 5%) was given intramuscularly for one week to prevent infection. One week after surgery, the rats were randomly arranged into sham and postmenopausal groups and were given different diets for 24 weeks as follows:

- Sham groups
  - Group I (control): rats received basal diet
  - Group II (250GP): rats received basal diet and supplemented with 250 mg/kg of *G. procumbens* extracts
  - Group III (500GP): rats received basal diet and supplemented with 500 mg/kg of *G. procumbens* extracts
  - Group IV (ATV): rats received basal diet and supplemented with 10 mg/kg of atorvastatin
- Postmenopausal (PM) groups
  - Group I (control): rats received 2% cholesterol diet fortified with 5HPO
  - Group II (250GP): rats received 2% cholesterol diet fortified with 5HPO and supplemented with 250 mg/kg of *G. procumbens* extracts
  - Group III (500GP): rats received 2% cholesterol diet fortified with 5HPO and supplemented with 500 mg/kg of *G. procumbens* extracts
  - Group IV (ATV): rats received 2% cholesterol diet fortified with 5HPO and supplemented with 10 mg/kg of atorvastatin

*G. procumbens* extract was given orally once daily using a gavage needle, and the supplementation was given for 24 weeks. Systolic blood pressure (SBP) was measured at the baseline and four-week intervals for 24 weeks using a noninvasive method while blood was collected through the orbital sinus at 0, 3, and 6 months. At each stipulated month, 6 mL of blood were collected in heparin and plain tubes, respectively. Blood in heparin was centrifuged for 10 min at 1000 ×*g* at 4°C to obtain plasma and erythrocyte, which were then washed three times with cold saline (4°C) and kept frozen at −80^o^C for biochemical analysis. Whereas, blood in plain tube was let to clot for 1 h before centrifuged at 9500 ×*g* for 10 min to obtain serum. Serum was kept frozen at −20°C for further analysis. Following 24 weeks of study, the animals were sacrificed with an overdose of diethyl ether and the thoracic aorta was isolated.

### 2.7. Measurement of Blood Pressure

A noninvasive method was used to measure systolic blood pressure (SBP) [23] using PowerLab data acquisition systems (AD Instruments, Castle Hill, NSW, Australia). A monitoring cuff was placed on the proximal end of the tail to detect changes in blood flow which occurred during occlusion or release of the cuff. The rats were put in an approximately body-sized plastic container prior to SBP measurement. This step ensured acclimation and faster SBP measurement. The animals were prewarmed for 15 min to increase blood flow to the tails. A minimum of five measurements were recorded, and the mean was used for analysis. The SBP was recorded every 4 weeks over 24 weeks of feeding period.

### 2.8. Aorta Histological Observation

Thoracic aorta specimens were cleaned and cut into 2.0 cm in diameter and mixed with optimal cutting temperature (OCT) compound onto a tissue mold. The frozen tissues were sectioned to 12 *μ*m thickness in the cryostat and were maintained at −20°C. The sectioned tissues were later mounted on clean glass slides and stained with Oil Red O (Sigma St. Louis, MO, USA) following previous protocol with some modifications [24]. In brief, each aorta tissues were rinsed in 60% 2-propanol for 3 min and then incubated in Oil Red O at 37°C for 40 min. Later, it was destained in 60% 2-propanol for 6 min and then mounted on a glass slide with aqueous mounting medium (Biomeda Corp.). Stained aortic tissues were examined and photographed microscopically by a contrast phase microscope (BX50, Olympus).

### 2.9. Measurement of Lipid Profile

The activity of cholesterol-LDL (LDH), cholesterol-HDL (HDL), total cholesterol (TC), and total triglycerides (TG) were quantified using an EnzyChrome kit (BioAssay Systems). The standards and samples were prepared following manufacturer's instruction with some modifications. Briefly, serum samples were vortexed, mixed, and centrifuged at 9500 ×*g* for 10 min. The supernatants were mixed with assay buffer and transferred into 1.5 mL Eppendorf tubes for each lipid profile measured. The mixtures were incubated at room temperature for 3 min, and the intensity of the coloured product (NADH) was measured at 340 nm using EnSpire Multimode Plate Reader (Perkin Elmer, USA).

### 2.10. Determination of Plasma Malondialdehyde Level

Plasma malondialdehyde (MDA) level was determined using high performance liquid chromatography (HPLC) with a photodiode array detector (Shimadzu, Japan) with some modifications [25]. Briefly, 50 *μ*L of plasma samples were mixed with 200 *μ*L of 1.3 M NaOH and incubated at 60°C for 30 min. The mixtures were left to cool at room temperature before adding 100 *μ*L of 35% of HClO_4_. Then, mixtures were centrifuged at 10000 ×*g* for 10 min, and the supernatants were transferred into 1.5 mL HPLC tubes. Subsequently, 5 mM of DNPH solution (50 *μ*L) was added into the mixtures and incubated for 30 min at room temperature. The samples (40 *μ*L) were then injected into the HPLC. The amount of MDA was expressed as the concentration of MDA in nmol per mL plasma.

### 2.11. Determination of Antioxidant Enzymes Activity

The activities of antioxidant enzymes superoxide dismutase (SOD, EC 1.15.1.1), glutathione peroxidase (GPx, EC 1.11.1.9), and catalase (CAT, EC 1.6.4.2) were determined using a commercial assay kit (Cayman USA) in a 96-well plate. All reagents were prepared according to the manual. Briefly, blood was collected in heparin tubes and centrifuged at 1000 ×*g* for 10 min at 4°C. Erythrocytes were isolated and lysed in four times of ice-cold HPLC-grade water. The lysed erythrocytes were centrifuged at 10,000 ×*g* for 15 min at 4°C. Subsequently, the supernatant was collected and diluted in 1 : 1000 of sample buffer. Absorbance of SOD activities was read at 460 nm, GPx activities at 340 nm, and CAT activities at 540 nm by the plate reader.

### 2.12. Statistical Analysis

Data obtained were expressed as mean ± standard deviation (SD). Differences between the experimental groups were analyzed using one-way analysis of variance (ANOVA) followed by Tukey HSD post hoc test. Differences were considered to be statistically significant for *p* < 0.05. All statistical analysis was carried out using SPSS for windows version 20.0.

## 3. Results

### 3.1. Liquid Chromatography-Tandem Mass Spectrometry

Atmospheric pressure chemical ionization (APCI) in the negative mode was used in LC-MS/MS analysis to tentatively identify the secondary metabolites present in the 80% ethanol extract of *G. procumbens*. Total ion chromatogram (TIC) of the components in *G. procumbens* extract and chlorogenic acid is shown in [Supplementary-material supplementary-material-1]. Identification and quantification of the compounds were performed using multiple reaction monitoring (MRM) detection-ion chromatogram at *m*/*z* values corresponding to the molecular weight of the identified compounds. The unprotonated molecular ions [M − H] were detected.  Table 1 shows the resultant retention times of the compounds along with their molecular ion peaks.

### 3.2. HPLC Analysis of 80% Ethanol Extract of *G. procumbens*

**Figure 1:**
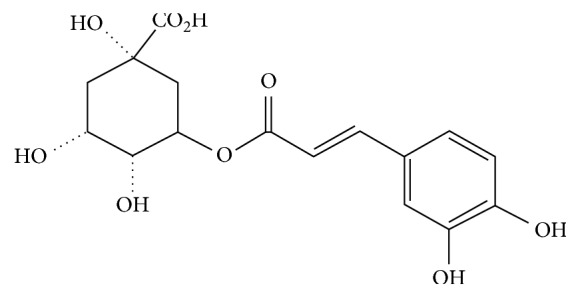
Chemical structure of chlorogenic acid.

**Figure 2:**
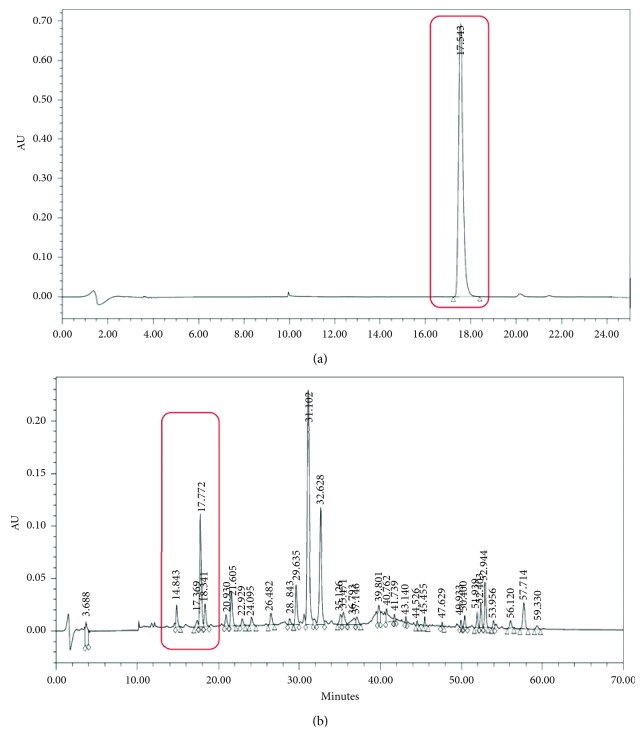
Representative RP-HPLC chromatograms of chlorogenic acid (standard) (a) and 80% ethanol extract of *G. procumbens* (b) for identification and quantification of chlorogenic acid in *G. procumbens* extract at 326 nm.

HPLC analysis on a reversed-phase (C-18) column with a gradient system of water-acetonitrile as the mobile phase was performed for qualitative and quantitative analysis of chlorogenic acid (Figure 1) in the 80% ethanolic extract of *G. procumbens* from GP1, GP2, and GP3 under the gradient conditions. The HPLC method was validated for its accuracy, specificity, linearity, and LOD-LOQ parameters. The linearity and accuracy of the method was established via the calibration curve of the reference standard, chlorogenic acid. Parameters such as correlation coefficient, *y*-intercept, slope of regression line, and residual sum of squares were evaluated. The calibration curve was established in triplicate. The regression equation was calculated based on the mean response vs the concentration where the regression value (*R*^2^ ≥ 0.99) indicated the method has good linearity. The results show the method has good specificity, indicated by the percentage of recovery which fell within the 90–110% range. The precision, repeatability, and reproducibility of the method were assessed by using intra- and interday experiments. The small values of the standard deviation of the retention times and RSD of the responses revealed the method was precise and repeatable. The limit of detection (LOD) and quantitation (LOQ) were then determined by using standard deviation of the response and the slope. The retention time (RT) of chlorogenic acid was found to be 17.5 min at 326 nm wavelength, based on RT comparison of standard compound and spiking technique (Figure 2). The extracts of GP1, GP2, and GP3 were found to contain chlorogenic acid at 4.21, 8.34, and 12.79 *μ*g/mL, respectively.

### 3.3. Body Weight

**Figure 3:**
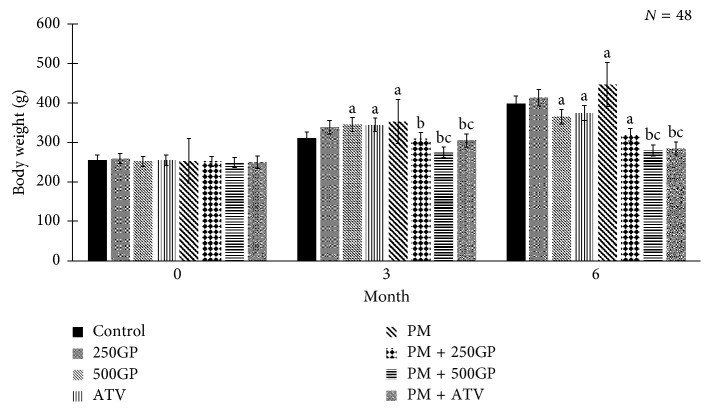
Effects of *Gynura procumbens* standardised extract supplementation on body weight in sham and postmenopausal (PM) groups at 0, 3, and 6 months of supplementation. Data are mean ± SD. ^a^Significant difference compared to control in the sham group (*p* < 0.05). ^b^Significant difference compared to the PM group (*p* < 0.05). ^c^Significant difference compared to the PM + 250GP group (*p* < 0.05).

An increasing pattern in body weight was observed in the postmenopausal group at months 3 and 6 compared to control in sham group (Figure 3). Body weight was reduced significantly with *G. procumbens* extract supplementation at 250 and 500 mg/kg starting at 3 months of supplementation compared to the postmenopausal group.

### 3.4. Histology of Thoracic Aorta

**Figure 4:**
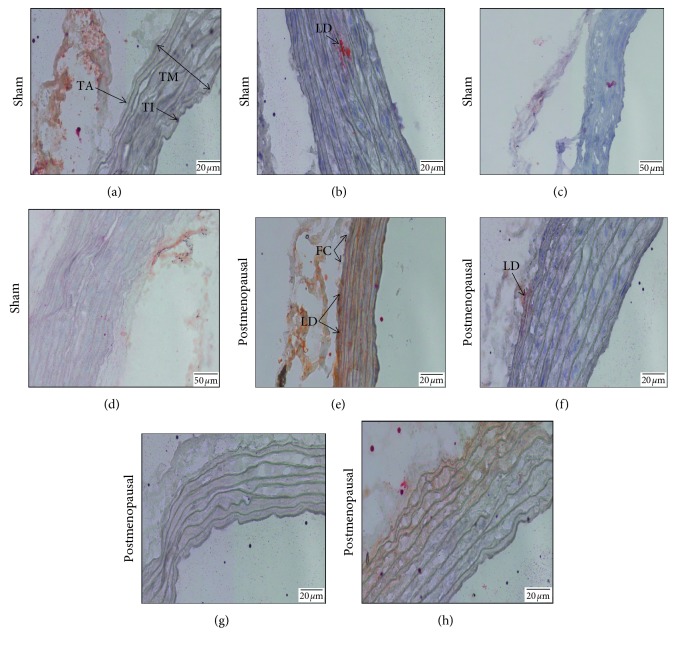
Effects of *Gynura procumbens* standardised extract supplementation on lipid droplets accumulation in the thoracic aorta in sham and postmenopausal (PM) groups stained with Oil Red O after 6 months of supplementation. (a) Control, (b) 250GP, (c) 500GP, (d) ATV, (e) PM, (f) PM + 250GP, (g) PM + 500GP, and (h) PM + ATV. Regions stained red with Oil Red O represent lipid droplets (LDs), and FC indicates foam cells. All cells were observed under the phase-contrast light microscope at 40x magnification.

Microscopic examination of the thoracic aorta in the postmenopausal group showed lipid droplets (cholesterol) accumulation (Figure 4(e))in the tunica media area, which was indicated by the red stain of Oil Red O reagent. Foam cells were observed in the tunica intima, resulting from the thickening of tunica media due to lipid accumulation. Supplementation with 250 and 500 mg/kg (Figures 4(f) and 4(g)) of body weight of the *G. procumbens* extract and statin was able to reduce the accumulation of lipid droplets in the postmenopausal group.

### 3.5. Blood Pressure

**Figure 5:**
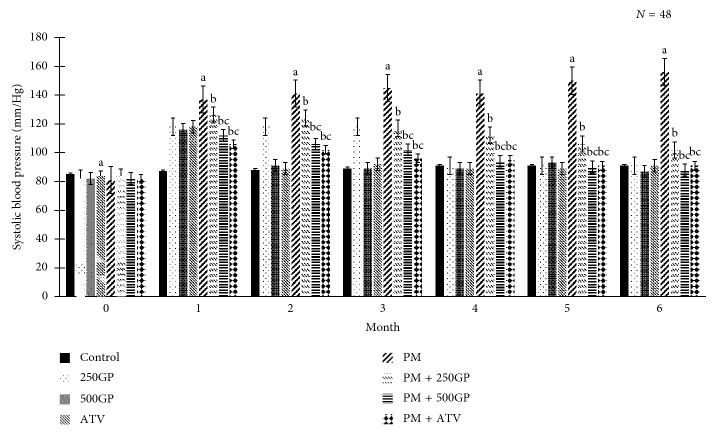
Effects of *Gynura procumbens* standardised extract supplementation on systolic blood pressure in sham and postmenopausal (PM) groups. Data are mean ± SD. ^a^Significant difference compared to control in the sham group (*p* < 0.05). ^b^Significant difference compared to the PM group (*p* < 0.05). ^c^Significant difference compared to the PM + 250GP group (*p* < 0.05).

As shown in Figure 5, blood pressure increased significantly (*p* < 0.05) in the postmenopausal group, and it was reduced with *G. procumbens* extract supplementation at 250 and 500 mg/kg of body weight. The blood pressure reduction was observed as early as in the first month of treatment with *G. procumbens* extracts. A similar effect was also observed with the supplementation of atorvastatin in the postmenopausal group.

### 3.6. Lipid Profile

**Figure 6:**
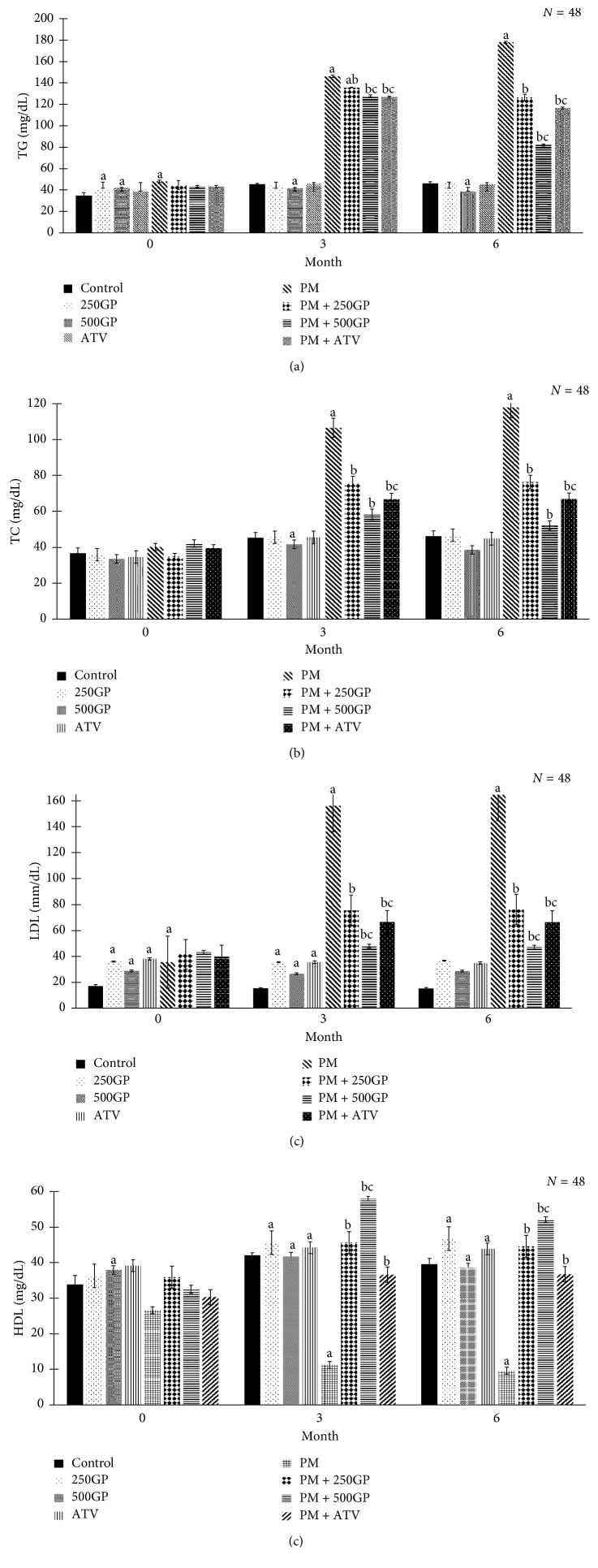
Effects of *Gynura procumbens* standardised extract supplementation on serum lipid profiles in sham and postmenopausal (PM) groups at 0, 3, and 6 months of study. Data are mean ± SD. (a) Total triglycerides, (b) total cholesterol, (c) low-density lipoprotein, and (d) high-density lipoprotein. ^a^Significant difference compared to control in the sham group (*p* < 0.05). ^b^Significant difference compared to the PM group (*p* < 0.05). ^c^Significant difference compared to the PM + 250GP group (*p* < 0.05).

Concentrations of plasma TG, TC, and LDL increased, and HDL decreased significantly (*p* < 0.05) in the postmenopausal group compared to the control (Figure 6). Supplementation of *G. procumbens* extract at 250 and 500 mg/kg of body weight and atorvastatin significantly (*p* < 0.05) reduced plasma concentrations of TG, TC, and LDL and increased plasma HDL. The reduction of TG, TC, and LDL and increase of HDL were better with 500 mg/kg of *G. procumbens* extract supplementation compared to 250 mg/kg of *G. procumbens* and atorvastatin group. Plasma lipid profiles in control groups were not affected by *G. procumbens* extract supplementation.

### 3.7. Plasma Malondialdehyde Level

**Figure 7:**
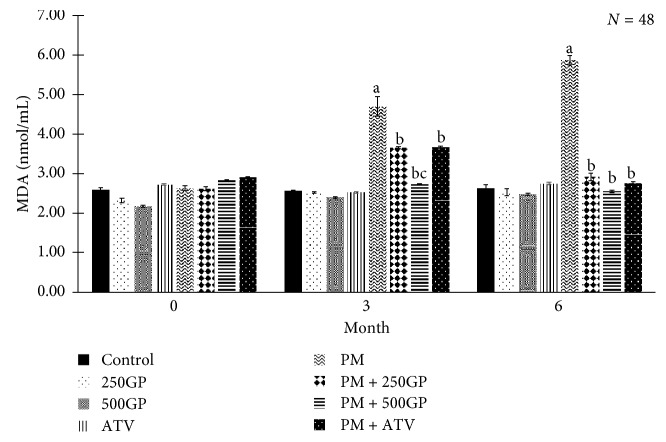
Effects of *Gynura procumbens* standardised extract supplementation on plasma malondialdehyde in sham and postmenopausal (PM) groups at 0, 3, and 6 months of study. Data are mean ± SD. ^a^Significant difference compared to control in the sham group (*p* < 0.05). ^b^Significant difference compared to the PM group (*p* < 0.05). ^c^Significant difference compared to the PM + 250GP group (*p* < 0.05).

Plasma MDA level was highly expressed in the postmenopausal group at months 3 and 6 compared to the control group. The level decreased in a dose-dependent manner with *G. procumbens* extract supplementation and atorvastatin in the postmenopausal group as presented in Figure 7. The reduction was observed in both doses of *G. procumbens* extract (250 and 500 mg/kg of body weight).

### 3.8. Antioxidant Enzymes Activity

**Figure 8:**
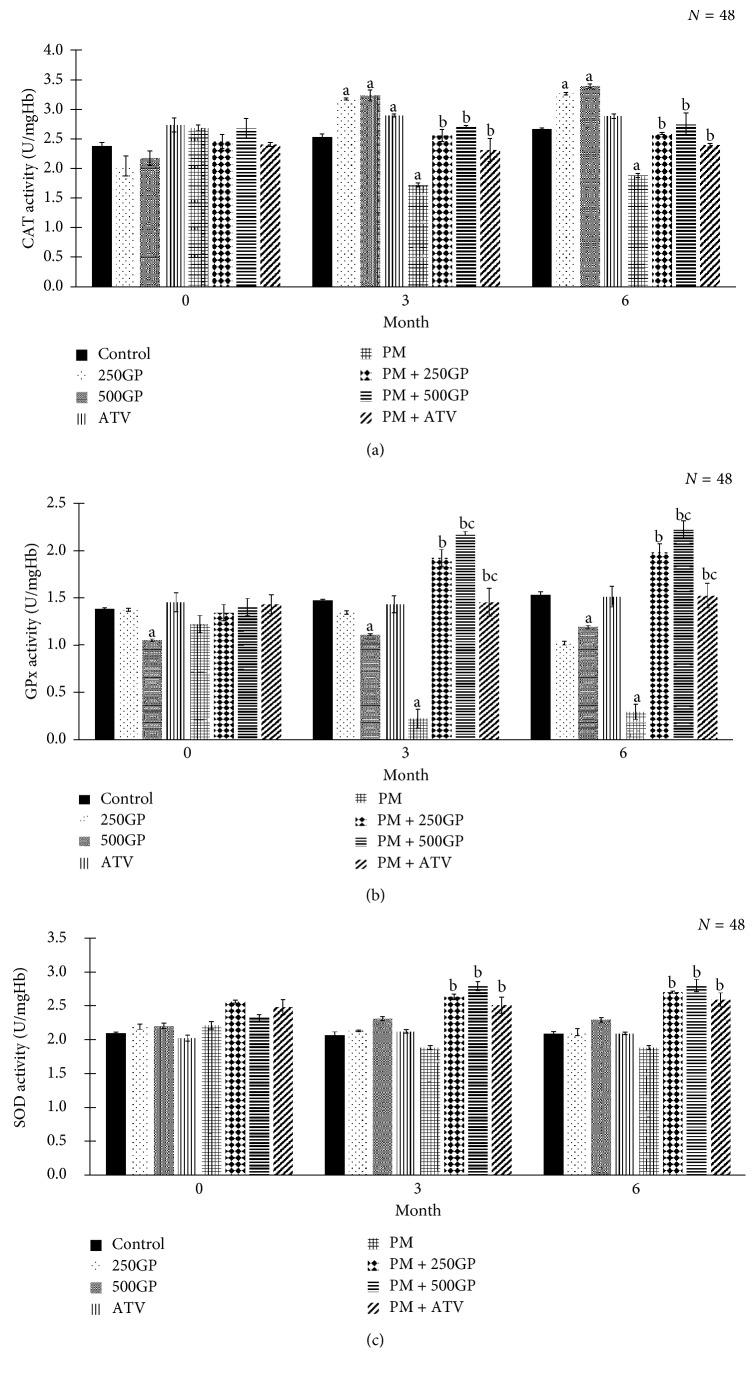
Effects of *Gynura procumbens* standardised extract supplementation on erythrocytes antioxidant enzymes activity in sham and postmenopausal (PM) groups at 0, 3, and 6 months of study. Data are mean ± SD. (a) CAT: catalase, (b) GPx: glutathione peroxidase, and (c) SOD: superoxide dismutase. ^a^Significant difference compared to control in the sham group (*p* < 0.05). ^b^Significant difference compared to the PM group (*p* < 0.05). ^c^Significant difference compared to the PM + 250GP group (*p* < 0.05).

*G. procumbens* standardised extract modulated antioxidant enzyme activities in erythrocytes as presented in Figure 8. The activities of GPx and CAT significantly decreased in the postmenopausal group at months 3 and 6. Following supplementation with *G. procumbens* extract at 250 and 500 mg/kg of body weight and atorvastatin, GPx, CAT, and SOD enzyme activities significantly increased (*p* < 0.05) compared to the postmenopausal group. *G. procumbens* extract supplementation maintained the SOD activity from month 3 until month 6.

## 4. Discussion

Numerous studies have been carried out to identify the antioxidant capacity of the *G. procumbens* extract. Many beneficial properties of *G. procumben*s have been revealed, which are attributed to the presence of bioactive compounds such as flavonoids, phenolic acids, alkaloids, glycosides, terpenoids, and sterols [26, 27]. Flavonoids, phenolic acids, and glycosides have been identified as the major components in the crude extract of *G. procumbens* leaves which are responsible for antioxidant activities involving nonenzymatic antioxidants, ferulic acid and sinapic acid [28]. The ability of *G. procumbens* extract to modulate antioxidant enzyme activities is supported by other studies [29]. In this study, the antioxidant properties of the 80% ethanol extract of the whole plant of *G. procumbens* were investigated. We identified the major phenolic contents which include chlorogenic acid, gallic acid, kaempferol, quercetin, and rutin in the 80% ethanol extract of *G. procumbens* by LC-MS/MS. Chlorogenic acid was chosen as the chemical marker for standardisation due to its reported cholesterol and other lipids lowering and cardiovascular protective effects. The 80% ethanol extracts of *G. procumbens* from GP1, GP2, and GP3 were standardised by HPLC. The extracts of GP3 which contained the highest amount of chlorogenic acid (12.79 *μ*g/mL) were used in this study.

Phenolic acids particularly chlorogenic acid (CGA), affect cholesterol metabolism by reducing the formation of micellar cholesterol in the digestive tract, and increasing bile flow, bile cholesterol, and bile acid concentration and excretion of cholesterol in the form of steroids [30, 31]. In previous study, CGA has been used to treat obese, hyperlipidaemic, and insulin resistant (*fa*/*fa*) in Zucker rats. It is shown that cholesterol and triacylglycerol were significantly decreased by 44% and 58%, respectively, when treated with CGA in plasma, the liver, and the spleen which suggested that *in vivo* CGA improves glucose tolerance and decreases plasma and liver lipids and other minerals pool distribution [32]. Other studies suggested that CGA inhibits fat absorption and activates fat metabolism in the liver with CGA treatment orally at 30 and 60 mg/kg/day for 14 days by reducing level of triglycerides in the liver [33]. A similar finding reported by Karthikesan et al. stated that CGA altered lipids, lipoproteins, and enzymes during lipid metabolism in STZ-nicotinamide-induced type 2 diabetes mellitus rats by decreasing the plasma and tissue (liver and kidney) lipids, cholesterol, triglycerides, free fatty acids, and low-density lipoproteins, respectively [34]. This finding might cause the blood pressure to reduce and return to normal range in atherosclerosis [35]. Chlorogenic acid lowered cholesterol and attenuated fatty liver by upregulating the gene expression of PPAR‐*α* in hypercholesterolemic rats induced with a high‐cholesterol diet [36]. Cho et al. [37] reported that chlorogenic acid showed antiobesity property and improved lipid metabolism in high-fat-diet-induced obese mice. This suggests that the bioactive compound in *G. procumbens* is an effective lipid-lowering agent and may reduce the risk of atherosclerosis with prolonged administration [38]. The flavonoid, eriocitrin, identified in this plant was reported to have powerful lipid-lowering activity as it improved dyslipidaemia and reduced lipid droplets in the liver of zebra fish [39]. Recently, eriocitrin has also reported to be identified in *G. procumbens* from China [40].

Different amounts of phenolic contents in *G. procumbens* extracts were obtained depending on the extraction method and solvent used. The highest amount of phenolics was obtained with methanol extraction followed by ethanol and water [41]. The different constituents identified in the present study and antioxidant potential when compared to previous reports may probably be due to extraction method and parts of the plant used [42]. Since the whole plant was used in this study, the number and amount of phenolic compounds may be denumbered by other plant constituents. In this study, most of the compounds were from polyunsaturated fatty acid (PUFA), and PUFA is known to have beneficial properties in CVD protection [43]. It has been reported that PUFA from natural sources has been linked with lower LDL cholesterol concentration in obese women but not from consumption of the plant sterols [44]. To the best of our knowledge, there has been no study on PUFA as a beneficial compound identified in *G. procumbens*.

In this study, histology analysis on the thoracic aorta using Oil Red O staining showed lipid droplets (cholesterol) accumulation (Figure 4(e)) in the tunica media of the postmenopausal rats. The absence of oestrogen, which has a cardioprotective effect, might influence the lipid accumulation by suppressing the expression of these two nuclear oestrogen receptors, ER*α* and ER*β*, in the endothelial cells and smooth muscle cells [45]. ER*α* and ER*β* most probably make lipid droplets more destabilised as oestrogens initiate new vessel formation [46]. As oestrogen levels decline, levels of LDL cholesterol increase and levels of HDL cholesterol decrease followed by the accumulation of fat and cholesterol in the arteries, thus contributing to atherosclerosis [47]. Apart from that, a high-fat diet which contained 2% cholesterol fortified with five-times-heated palm oil (5HPO) was used in promoting atherosclerotic condition [48, 49]. Accumulation of lipid droplets is normal in a tissue. However, excessive lipid droplet accumulation within tissues can be an indicator of metabolic deficiency or pathogenesis [50]. For instance, excessive accumulation of lipids in liver cells (steatosis) can lead to cellular dysfunction. At the onset of atherosclerosis, macrophages engulf oxidized LDL, develop into foam cells, and contribute to artery narrowing due to lipid droplet accumulation. *G. procumbens* extract at both doses (250 and 500 mg/kg) has great potential in lipid droplet reduction. Our study also observed the effect of statin treatment as a positive control (Figure 4(h)) in regard to lipid droplet reduction. It was shown that *G. procumbens* extract at 500 mg/kg has a better effect in reducing lipid accumulation in thoracic aorta compared to statin.

Our results indicate that increased body weight in the postmenopausal group at months 3 and 6 was in relation to the prolonged feeding of high-fat diet. This is also supported in another study [51] where diet enriched with 2% cholesterol and 5HPO increased food intake by rats. On the other hand, a different finding was presented [52], which stated that when palm oil is repeatedly used for deep-frying, oxidation would degrade oil quality and alter the taste, texture, and odour, which may influence food intake. In addition, high-fat diet which contains 2% cholesterol fortified with 5HPO has been used in promoting atherosclerotic condition [48, 49]. Supplementation with *G. procumbens* standardised extract was able to reduce body weight in a dose-dependent manner. This might be due to the flavonoids and phenolic constituents in *G. procumbens*. This finding is in agreement with an earlier study which reported that these compounds are naturally occurring bioactive compounds that represent a constituent of vegetables, beyond calorie and macronutrient contents, that could potentially influence body weight [53].

The *G. procumbens* standardised extract exerted strong, dose-dependent, and blood pressure‐lowering effects in postmenopausal rats which was seen as early as the first month of supplementation. Prolonged supplementation with low and high doses of the *G. procumbens* standardised extract gave positive changes in blood pressure. These results are consistent with previous studies in spontaneously hypertensive rats, which showed that *G. procumbens* inhibits calcium channels, which leads to vasodilatation [54, 55]. The decrease in blood pressure may be attributed from the effect of *G. procumbens* extract on the endothelial cell function by lowering the total peripheral resistance of the blood vessels [56]. In the present study, the elevation of blood pressure in the postmenopausal group might be due to high cholesterol level, but with the *G. procumbens* supplementation, both the blood pressure and TC were reduced. We suggested that the phenolic acid constituent in the *G. procumbens* extract might have an effect towards the cholesterol metabolism. A previous study reported that phenolic acids were able to reduce TC and LDL and increase HDL [57]. Another study also reported that serum TC and TG in hyperlipidaemic rat were reduced with *G. procumbens* supplementation [58].

Palm oil that has been heated five times undergoes chemical changes that lead to the production of hydrolysis, oxidation, and polymerization at molecular level [56]. The products produced from the chemical changes process are volatile and nonvolatile agents such as hydroperoxides, aldehydes, oxidative dimers, and oxidized triacylglycerols [59, 60] which could lead to cellular damage by reacting with various biomolecules such as proteins, nucleic acids, or lipids. The aggravation of the cellular damage would promote lipid peroxidation and elevate oxidative stress and hence lead to hyperlipidaemia. Diets high in oxidized LDL have been associated with the elevation of oxidative stress, which is reflected by the increment of oxLDL and plasma MDA levels and also the increase in blood pressure. High level of oxLDL and lipid peroxidation has great implication on ROS production, contributing to early atherosclerosis [61]. OxLDL is engulfed by macrophages and accumulates in the intima lining of the arterial wall and reduces blood flow [62]. *G. procumbens* extract supplementation was able to decrease the MDA plasma level. MDA is the end product of lipid peroxidation. This might be due to the phenolic content in *G. procumbens* which inhibited oxidation of LDL through detoxification of the ROS and thus reducing oxidative stress [63].

ROS is detoxified or inactivated by antioxidant enzyme activities such as SOD, GPx, and CAT [64]. High amount of free radicals alters the function of the body's defence system. *G. procumbens* extract supplementation increased the activities of antioxidant enzymes (CAT, GPx, and SOD) in this study. The flavonoids and phenolic acids of *G. procumbens* have been shown to be able to protect erythrocytes against oxidative damage by modulating the activities of the antioxidant enzymes [65]. These compounds act as free-radical scavengers to stabilise the defence system [66]. Apart from that, PUFA might also play a role in reducing LDL cholesterol level and may have cardioprotective properties [67]. PUFA reduces LDL particle size rather than the number of LDL through the modification of the LDL composition by increasing apolipoprotein B and reducing lipoprotein level. In our study, PUFA was identified but has not yet been conclusively elucidated.

Other studies have reported that phytochemicals may be hormetic where they have beneficial effects at low doses but are toxic in high doses [57]. We suggested *G. procumbens* extract supplementation at 500 mg/kg body weight is the best dose that could give beneficial effect towards atherosclerosis, but the actual mechanism needs to be further explored.

## 5. Conclusions

In conclusion, *G. procumbens* extract supplementation has the ability to reduce lipid oxidation through the modulation of antioxidant enzyme activity and prevent atherosclerosis. However, there is still limited knowledge regarding the underlying mechanisms of action and exact chemical constituents involved. Further research elucidating the mechanisms underlying the biological activities is needed for development of standardised drugs or herbal products for use in treating or preventing atherosclerosis.

## Figures and Tables

**Table 1:** Retention times (RTs) and molecular ion peak [M − H]^−^ of the tentative compounds present in *Gynura procumbens*.

Identified compound	RT (min)	Molecular ion [M − H]^−^ (*m*/*z*)
(1) Unidentified	0.9	244.990
(2) Caffeic acid	—	179.030
(3) Trimethyl gallic acid glucuronide	—	387.000
(4) 5-O-(E)-Caffeoyl-galactaric acid	4.13	371.000
(5) Chlorogenic acid	4.33	609.520
(6) Unidentified	4.53	496.190
(7) Rutin	4.81	609.160
(8) Quercertin	4.91	300.923
(9) Neochlorogenic acid	4.99	—
(10) Nicotiflorin	5.02	593.000
(11) Astragalin	5.12	447.000
(12) Kaempferol	5.79	284.916
(13) 4-0 Methyl gallic acid sulphate or 3-0 methyl gallic acid sulphate	—	263.070
(14) Oxooctadecanoic acid isomer	—	298.170
(15) Unidentified	5.98	—
(16) Genkwanin isomer	—	572.290
(17) Eriocitrin	—	595.280
(18) 15,16-Dihydroxy-9Z, 12Z-octadecadienoic acid	7.08	311.200
(19) Unidentified	7.59	—
(20) Oxooctadecanoic acid derivative	—	312.190
(21) Oxooctadecanoic acid	9.52	297.300
(22) Unidentified	10.83	817.000

## Data Availability

The data used to support the findings of this study are included within the article.

## Supplementary Materials

Supplementary Materials Figure S1: LC-MS/MS full chromatograms of *G. procumbens* (A) and chlorogenic acid (B).
