# Supplementary material for: Gynura procumbens Standardised Extract Reduces Cholesterol Levels and Modulates Oxidative Status in Postmenopausal Rats Fed with Cholesterol Diet Enriched with Repeatedly Heated Palm Oil
Source: Evid Based Complement Alternat Med. 2019 Sep 23;2019:7246756. doi: 10.1155/2019/7246756 (PMC6778942; doi:10.1155/2019/7246756)
Supplement: Supplementary Materials — Figure S1: LC-MS/MS full chromatograms of G. procumbens (A) and chlorogenic acid (B). [file 7246756.f1.pdf]

Supplementary Materials

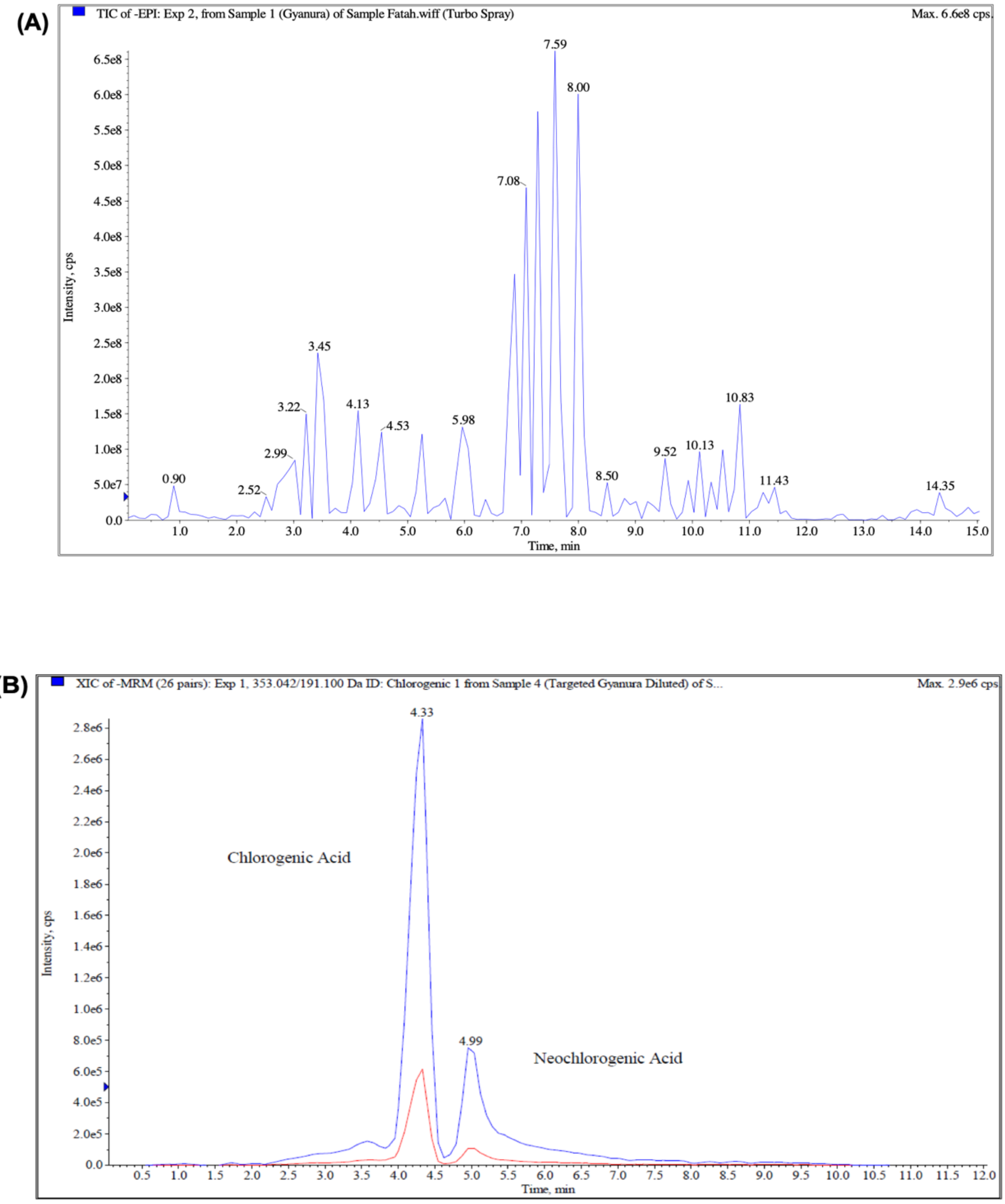

Figure S1. LC–MS/MS full chromatograms of *G. procumbens* (**A**) and chlorogenic acid (**B**).
